# Prognostic value of platelet-to-basophil ratio (PBR) in patients with primary glioblastoma

**DOI:** 10.1097/MD.0000000000034506

**Published:** 2023-07-28

**Authors:** Chao Yang, Jian Xu, Jie Wang, Zhiqiang Li, Qiong Yao

**Affiliations:** a Department of Neurosurgery, Zhongnan Hospital of Wuhan University, Wuhan, China; b Department of Pathology, Zhongnan Hospital of Wuhan University, Hubei, China.

**Keywords:** glioblastoma, nomogram, platelet-to-basophil ratio (PBR), prognosis, survival

## Abstract

Inflammation is strongly associated with cancers. Our research explored the prognostic role of hematological inflammatory indicators in patients with glioblastoma (GBM). Hundred patients were incorporated finally, and we determined the best cutoff values of these blood markers through X-tile first to perform further survival tests. The prognostic role of peripheral blood cell count and corresponding ratios were assessed using the Kaplan–Meier curve and Cox models to identify variables with independent prognostic significance. Then, variables with independent predictive values were incorporated in constructing the nomogram model to realize quantitative prediction for survival. Nomograms were evaluated using Harrell concordance index, receiver operating characteristic curves, and decision curve analysis to assess clinical benefit. Multivariate analysis indicated that a higher platelet-to-basophil ratio (PBR) (>4575) (hazard ratio = 1.819, 95% confidence interval: 1.110–2.980, *P* = .018) was associated with poorer overall survival in GBM patients. Based on the PBR nomogram, the predictive accuracy was moderate (concordance index = 0.844, area under curve = 0.632). The PBR could serve as a prognostic biomarker for overall survival in patients with GBM, and nomogram models incorporating the PBR could facilitate comprehensive preoperative survival assessment.

## 1. Introduction

In the brain, glioblastoma (GBM), the most common malignant tumor, remains a poor prognosis,^[1]^ though major advances have been made recently in resection safety, chemotherapy, and tumor-treating fields.^[2,3]^ As a result, finding prognostic biomarkers for GBM patients is extremely important. With the remarkable advances of whole-genome sequencing technologies, many molecular biomarkers with diagnostic or predictive value have been identified in glioma, and molecular type has also been added to the classification of glioma in the revised 2016 World Health Organization classification of the central nervous system firstly. Isocitrate dehydrogenase 1 (IDH 1) and O6-methylguanine-DNA methyltransferase (MGMT) were the most commonly used molecular biomarkers, and their diagnostic or prognostic value of them in glioma had been widely reported.^[4]^ The cost and complexity of the test technology have limited their widespread application, and these molecular markers can only be obtained via postoperative testing. To this end, developing preoperative biomarkers with lower cost and non-invasion is necessary.

The dynamic relationship between tumor cells and the immune microenvironment plays a crucial role in cancer pathogenesis.^[5]^ In different types of malignant tumors, hematological inflammatory markers, easily obtained through blood tests, have been investigated as prognostic factors. It was found that lymphocytes, neutrophils, and platelets constituted the majority of peripheral blood cells; their levels were associated with the state of the immune system’s inflammation. There has been substantial research into the prognostic significance of blood cells and their ratios, such as neutrophil-to-lymphocyte ratio,^[6,7]^ lymphocyte-to-monocyte ratio,^[8,9]^ platelet-to-lymphocyte ratio^[10,11]^ and systemic immune-inflammation index.^[12]^ However, eosinophils and basophils were often neglected in evaluating prognosis in patients with glioma.

Interestingly, atopic diseases are negatively associated with susceptibility to glioma and are associated with improved prognosis in patients with glioma.^[13,14]^ Moreover, essential roles of basophils and eosinophils in the pathobiology of atopic disease have been identified. Therefore, this study aimed to explore the prognostic value of eosinophils and basophils in patients with GBM.

## 2. Materials and methods

### 2.1. Inclusion and exclusion criteria

The study included 100 adult GBM patients from January 2016 through June 2020. We included patients over 18 years of age at diagnosis who had pathological confirmation of their diagnoses and could provide data about their clinical characteristics and blood samples. There were 4 exclusion criteria: Patients receiving radiotherapy and chemotherapy before surgery were excluded from consideration (including any oral corticosteroids, like prednisone and hydrocortisone as a substitute therapy or intravenous steroids to relieve cerebral edema before surgery); Recurrent GBM; Patients accompanied with a prior history of cancers; or Perioperative deaths occurred in patients. The Helsinki Declaration was followed throughout.^[15]^ The follow-up ended on September 30, 2021.

### 2.2. Ethical approval

The medical ethics committee at our hospital approved the present study (No. 2019048).

### 2.3. Collection of data

A few variables were collected, including sex, age, locations of tumor, the characteristics of Karnofsky performance status (KPS), the mutation of IDH1 and the methylation of MGMT promoter, total resection (GTR, 100%), subtotal resection (STR, <100%), and full chemoradiotherapy postoperatively. Preoperative blood tests were also reviewed, including counts of neutrophils, lymphocytes, platelets, eosinophils, and basophils. Using these data, the corresponding ratios, EBR (eosinophil-to-basophil ratio), NER (neutrophil-to-eosinophil ratio), NBR (neutrophil-to-basophil ratio), LER (lymphocyte-to-eosinophil ratio), LBR (lymphocyte-to-basophil ratio), PER (platelet-to-eosinophil ratio) and platelet-to-basophil ratio (PBR) (platelet-to-basophil ratio) were also calculated. An individual’s overall survival (OS) is the range between when they are operated on until they die with all-cause or until their last follow-up. They were followed by outpatient visits or by phone.

### 2.4. Statistical analysis

Interquartile range and median are nonparametric measures used to analyze nonnormal distributions, and mean ± standard deviation defines normal distribution, respectively. Chi-square tests are used to compare categorical variables between groups based on frequency (percentages). The receiver operating characteristic curve identified the optimal eosinophil and basophil cutoff values, and the rest indicators were obtained using X-tile software (version 3.6.1). We performed Kaplan–Meier survival curves and interpreted the results using the log-rank test (R survminer package). By using the R survival package, univariate and multivariate Cox proportional hazard regression analyses were performed to assess the independent prognostic significance of these markers. Nomograms were created using R rms to predict 2-year survival probability based on variables with independent values. Drawing the calibration figure to analyze the consistency between the predicted and observed values. And time-dependent receiver operating characteristic (ROC) curve analysis was used to evaluate the discrimination of the nomogram. Statistical analysis was performed with R software (version 4.0.2, Vienna, Austria) using 2-sided *P* values, with a *P* value of <.05 defining statistical significance.

## 3. Results

### 3.1. Characteristics of clinical and pathological manifestations

As shown in Table 1, all incorporated patients were males (60%) and females (40%), with a mean age of 57.2 years. The proportion of GBM patients who underwent STR was 58.0%, and 59.0% received chemoradiotherapy administered by Stupp postoperatively. Patients with IDH1 mutation or MGMT methylation accounted for 4 (4.0%) cases and 57 (57.0%), respectively. The number of patients taking other medications, like antibiotics and antidiabetics, was 28 (28%) in the last 3 months. Besides, 15 (15%) and 8 (8%) patients also have hypertension and diabetes, respectively.

**Table 1:** Clinical and pathological characteristics of patients.

Characteristic	PBR ≤ 4575	PBR > 4575	*P* value
n	29	71	
Sex, n (%)			.964
Female	11 (37.9%)	29 (40.8%)	
Male	18 (62.1%)	42 (59.2%)	
Location, n (%)			.126
Frontal	11 (37.9%)	15 (21.1%)	
Multiple	5 (17.2%)	14 (19.7%)	
Other	2 (6.9%)	17 (23.9%)	
Parietal	5 (17.2%)	6 (8.4%)	
Temporal	6 (20.7%)	19 (26.8%)	
Hypertension, n (%)			.926
Yes	5	10	
No	24	61	
Diabetes, n (%)			.884
Yes	2	6	
No	27	65	
KPS, n (%)			.309
>60	21 (72.4%)	42 (59.2%)	
≤60	8 (27.6%)	29 (40.8%)	
Chemoradiotherapy, n (%)			.284
No	9 (31.0%)	32 (45.1%)	
Yes	20 (69.0%)	39 (54.9%)	
Resection, n (%)			.138
GTR	16 (55.2%)	26 (36.6%)	
STR	13 (44.8%)	45 (63.4%)	
IDH1, n (%)			1.000
Wildtype	28 (96.6%)	68 (95.8%)	
Mutant	1 (3.4%)	3 (4.2%)	
MGMT, n (%)			.666
Yes	18 (62.1%)	39 (54.9%)	
No	11 (37.9%)	32 (45.1%)	
Medications, n (%)			.582
Yes	7 (24.1%)	21 (29.6%)	
No	22 (75.9%)	50 (70.4%)	
Age, median (IQR)	62 (53, 69)	58 (48.5, 63.5)	.259
OS, median (IQR)	13.6 (8.3, 20.5)	8.8 (5.25, 16.6)	0.013

GTR = gross total resection, IDH = isocitrate dehydrogenase, IQR = interquartile range, KPS = Karnofsky performance status, MGMT = O^6^-methylguanine-DNA methyltransferase, OS = overall survival, PBR = platelet-to-basophil ratio, STR = subtotal resection.

### 3.2. A correlation between hematological indices and survival in GBM patients

**Figure 1.**
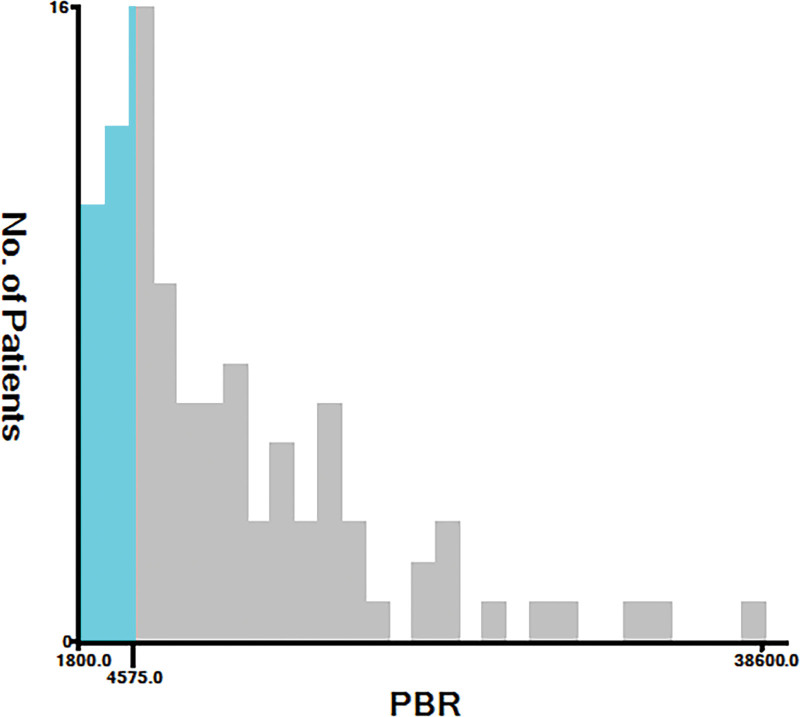
cutoff of PBR in patients with glioblastoma. PBR = platelet-to-basophil ratio.

**Figure 2.**
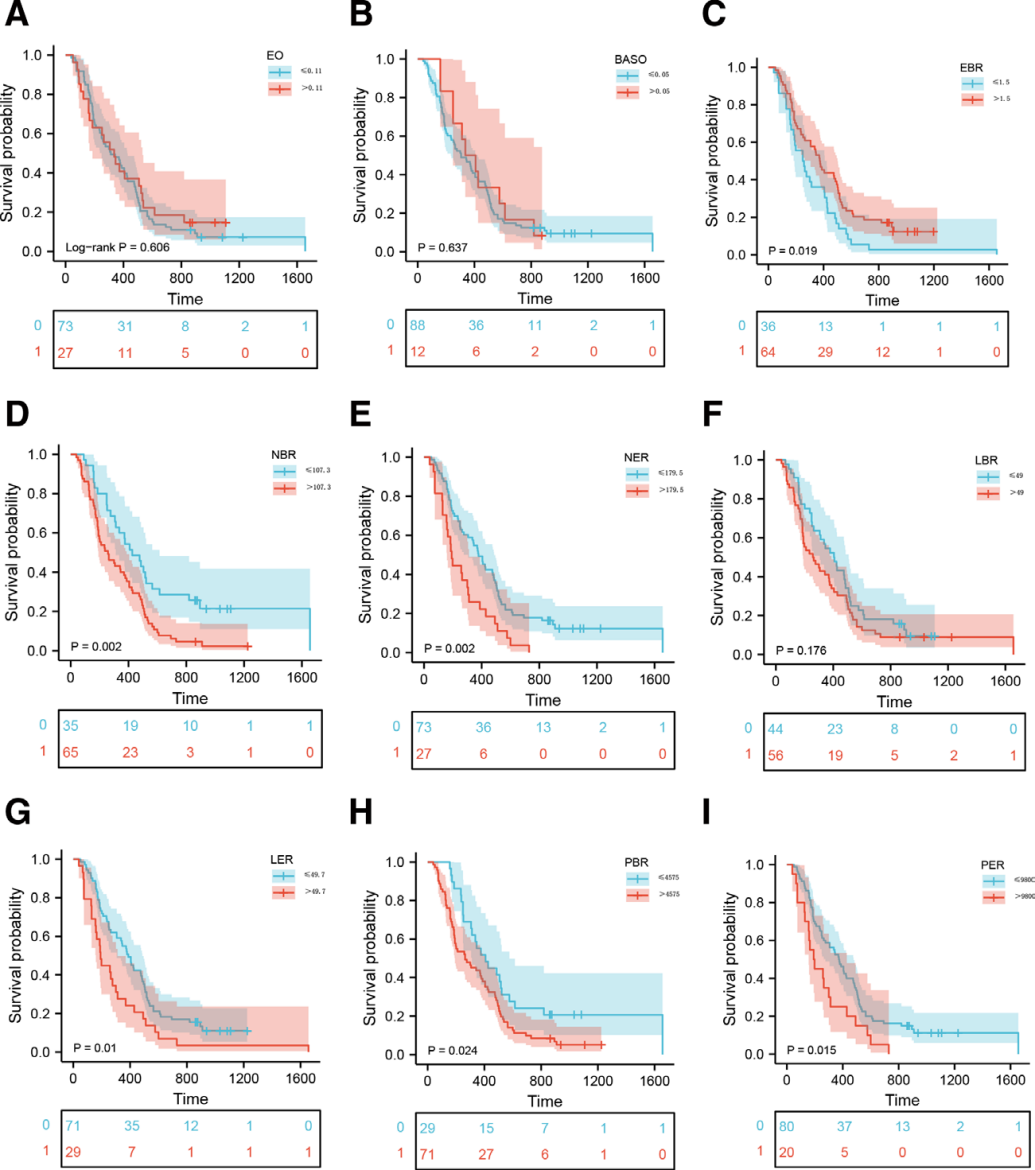
Kaplan–Meier survival curves of GBM patients based on the cutoff values of eosinophil (A), basophil (B), EBR (C), NBR (D), NER (E), LBR(F), LER (G), PBR (H), and PER (I). GBM = glioblastoma, PBR = platelet-to-basophil ratio.

The optimal cutoffs for each peripheral marker were obtained using X-tile software or a ROC curve. As shown in Figure 1 and Figure S1, Supplemental Digital Content, http://links.lww.com/MD/J379, the cutoff values for EBR, NER, NBR, LER, LBR, PER, and PBR were 1.5, 179.5, 107.3, 49.7, 49, 9800, and 4575, respectively. The cutoff values for eosinophil and basophil were 0.11 and 0.05, respectively (10^9^ cells/L). According to the cutoff value of each marker, the patients were divided into 2 groups. Based on survival analysis, it was found that among GBM patients, a higher NER (*P* = .002), NBR (*P* = .002), LER (*P* = .01), PER (*P* = .015), and PBR (*P* = .024) associating with a worse outcome, however those with a higher EBR (*P* = .019) had a better OS (Fig. 2). No significant relationships were observed between eosinophil (*P* = .606), basophil count (*P* = .637), or LBR (*P* = .176) and OS (Fig. 2). The multivariate analysis of Table 2 revealed that only PBR was independent of prognostic significance besides postoperative chemoradiotherapy, KPS, and resection. Univariate analysis showed that age, EBR, NER, NBR, LER, and PER were significant variables related to survival outcome, but they did not conduct independent prognostic value in multivariate analysis. Moreover, no significant relationships were observed between medications (like antibiotics and antidiabetics) and OS (Table 2).

**Table 2:** Univariate and multivariate analyses of OS in GBM cohorts.

Characteristics	Univariate analysis		Multivariate analysis	
	Hazard ratio (95% CI)	*P* value	Hazard ratio (95% CI)	*P* value
Sex	1.009 (0.661–1.540)	.967	/	/
Age (>57/≤57)	1.027 (1.006–1.048)	.011	1.018 (0.998–1.038)	.077
Location	1.078 (0.934–1.244)	.306	/	/
KPS (>60/≤60)	0.883 (0.862–0.905)	<.001	0.908 (0.883–0.934)	<.001
Chemoradiotherapy (yes/no)	0.098 (0.054–0.176)	<.001	0.437 (0.210–0.908)	.027
Resection (GTR/STR)	0.174 (0.107–0.282)	<.001	0.433 (0.240–0.781)	.005
IDH1 (mutant/wild)	0.771 (0.282–2.106)	.612	/	/
MGMT (yes/no)	0.713 (0.467–1.088)	.117	/	/
Medications (yes/no)	0.726 (0.462–1.139)	.163		
EO (>0.11/≤0.11)	0.881 (0.548–1.417)	.602	/	/
BASO (>0.05/≤0.05)	0.859 (0.456–1.617)	.637	/	/
EBR (>1.5/≤1.5)	0.599 (0.389–0.920)	.019	0.746 (0.470–1.185)	.214
NER (>179.5/≤179.5)	2.048 (1.293–3.244)	.002	1.452 (0.877–2.403)	.148
NBR (>107.3/≤107.3)	2.035 (1.286–3.220)	.002	1.450 (0.892–2.355)	.134
LER (>49.7/≤49.7)	1.799 (1.148–2.821)	.010	1.534 (0.942–2.499)	.085
LBR (>49/≤49)	1.335 (0.879–2.028)	.176	/	/
PER (>9800/≤9800)	1.860 (1.125–3.074)	.015	1.091 (0.629–1.892)	.756
PBR (>4575/≤4575)	1.729 (1.074–2.781)	.024	1.819 (1.110–2.980)	.018

BASO = basophil, CI = confidence interval, EBR = eosinophil-to-basophil ratio, EO = eosinophil, GBM = glioblastoma, GTR = gross total resection, HR = hazard ratio, IDH = isocitrate dehydrogenase, KPS = Karnofsky performance status, MGMT, O^6^-methylguanine-DNA methyltransferase, NBR = neutrophil-to-basophil ratio, NER = neutrophil-to-eosinophil ratio, LBR = lymphocyte-to-basophil ratio, LER = lymphocyte-to-eosinophil ratio, OS = overall survival, PBR = platelet-to-basophil ratio, PER = platelet-to-eosinophil ratio, STR = subtotal resection.

### 3.3. Nomograms in GBM patients for predicting the survival rate

**Figure 3.**
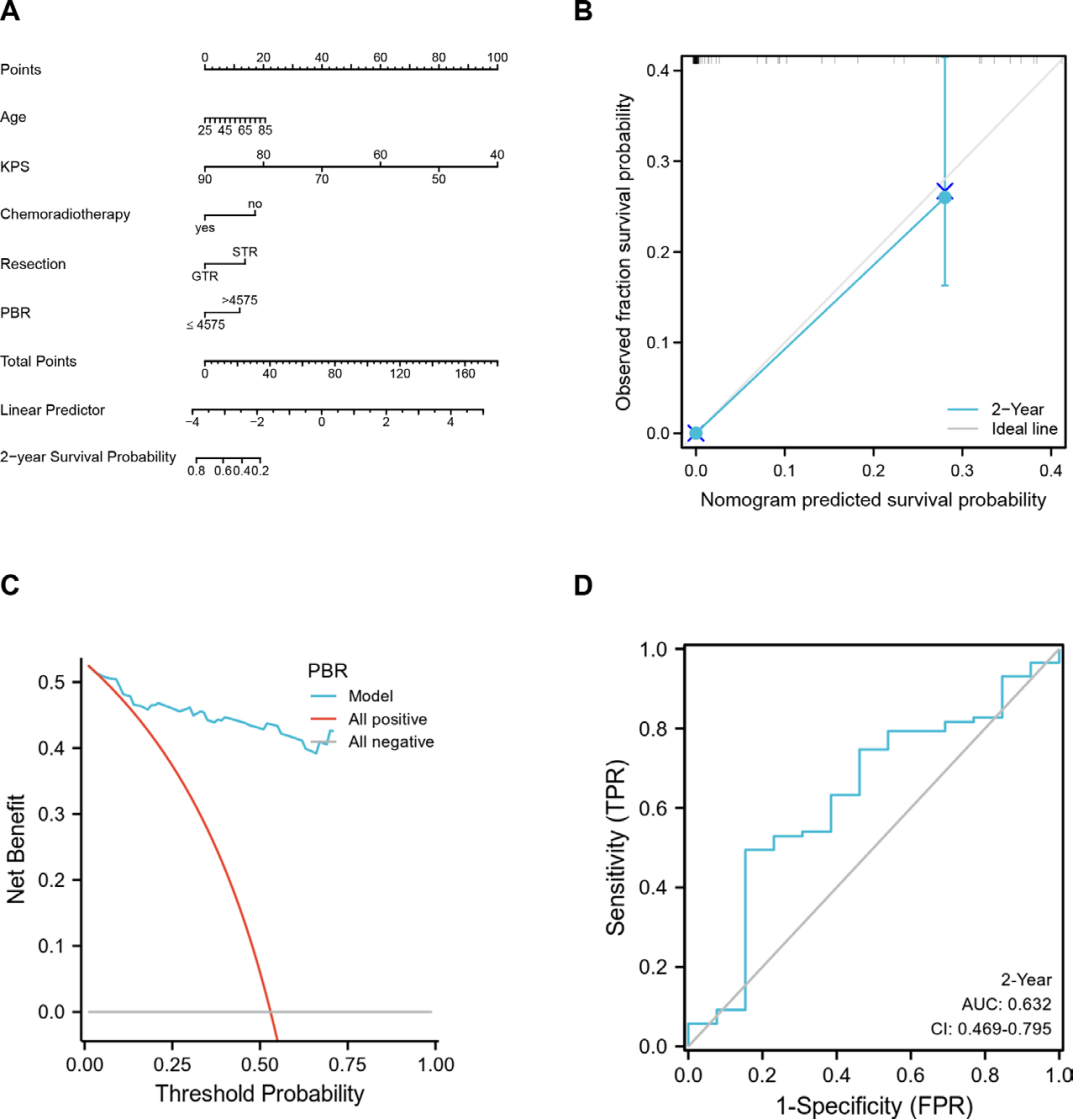
In the nomogram (A), each variable was assigned a different score as shown on the highest scale, and the total score for all items yielded a quantitative prediction of a 2-year survival rate, with a higher score leading to a worse prognosis. In the calibration plot (B), the dotted line represents the ideal prediction, and the full red line represents the prediction of the nomogram, (C) decision curve analysis of PBR for predicting OS in GBM patients, and (D) Time-dependent ROC curve analysis of PBR for predicting OS in GBM patients. GBM = glioblastoma, OS = overall survival, PBR = platelet-to-basophil ratio, ROC = receiver operating characteristic curve.

We determined several independent prognostic markers through multivariate regression analysis, including KPS, standard postoperative chemoradiation, PBR, and the extent of resection. Considering that age showed a tendency to correlate significantly with OS. As a result, age was also incorporated. In order to estimate the importance of these variables in predicting the 2-year survival probability for patients with GBM, a nomogram was constructed (Fig. 3A). As shown by the nomogram, KPS significantly contributed to survival, followed by age, chemoradiotherapy, tumor resection, and PBR. It had a concordance index of 0.844. Compared to the ideal model, the calibration plot with bootstrapped method performed better (Fig. 3B). Using decision curve analysis analysis, the nomogram was demonstrated to be clinically valuable and better able to discriminate (Fig. 3C). Moreover, the nomogram also had a moderate value of area under the curve (AUC = 0.632) based on the time-dependent ROC curve (Fig. 3D).

## 4. Discussion

Inflammation was closely associated with many biological systems and disease processes, including the initiation and progression of cancers.^[16,17]^ Patients with cancer, including those with gliomas, have been reported to have a higher survival risk if they have peripheral inflammation. The present study showed that higher PBR values were associated with worse outcomes in patients with GBM, and the prediction model that combined PBR and nomograms had good accuracy.

The mechanism of the effect of PBR on prognosis had not been fully elucidated. Leukocytes could cross the blood-brain barrier during the development of tumor.^[18]^ The migration of basophils to the TME has also been demonstrated in primary human tumors.^[19,20]^ It is reported that basophils in tumors can enhance the recruitment of tumor-specific CD8^+^ T cells to tumors by producing chemokines CCL3 and CCl4.^[21]^ As a rare peripheral leukocyte, the role of basophils has been neglected in previous research. Basophils had the potential to pro-tumor by inducing Th2 differentiation.^[22]^ However, the IgE receptor, FcεRI had a high affinity to human eosinophils^[23,24]^ and basophils.^[25]^ Subsequently, IgE-FcεRI complex on effector cells had the potential of phagocytosis to tumor cells through antibody-dependent cellular cytotoxicity and antibody-dependent cellular phagocytosis.^[26,27]^ In addition, it has been reported that basophils have direct antitumor effects and can release inflammatory cytokines (TNF- α, IL-6 and IL-1 β) to induce tumor cell apoptosis.^[28,29]^ Therefore the basophils in cancer may become a potential therapeutic target.^[30]^ A review published recently demonstrated that basophils and the activated markers of basophils were present in the tumor microenvironment, and high levels of basophils in the tumor around correlated with a better outcome.^[31]^ There are several ways in which elevated platelets may accelerate the proliferation, angiogenesis, and dissemination of tumor cells in patients with malignant tumors, one of which is through some released factors, like vascular endothelial growth factor and platelet-derived growth factor.^[32,33]^

Previous reports have determined the relationship between basophils and the survival outcome of pancreatic cancer,^[34]^ colorectal cancer,^[19]^ and non-small cell lung cancer.^[35]^ A recent report found that the ratio of basophils negatively associate with the number of visceral lung metastatic sites in tumor-bearing mice.^[36]^ Hadadi et al^[37]^ reported that higher levels of baseline basophils were associated with worse survival in patients with prostate cancer. Notably, a recent study by Zheng et al^[38]^ demonstrated that the baseline preoperative basophil count was independently correlated with progression-free survival in GBM patients.^[38]^ However, the present study first combined basophil and platelet and found an independent prognostic value of PBR in patients with GBM.

It is widely accepted that nomograms can be used in oncology research to calculate numerical estimates of individual clinical events.^[39]^ Resection, KPS, standard Stupp chemoradiotherapy regimen, and PBR were independent prognostic markers incorporated to construct a nomogram in the current research based on multivariate analysis; besides, considering the age showed a tendency to correlate with OS significantly. As a result, age was also incorporated, and clinical benefits and predictive accuracy of the nomogram were modest.

Additionally, the present study has some limitations. First, its retrospective nature could have led to selection bias. Second, we included a relatively small number of GBMs. Therefore, It is necessary to conduct prospective studies with more samples to clarify the results.

## 5. Conclusion

The PBR could serve as a prognostic biomarker for OS in patients with GBM, and nomogram models incorporating the PBR could facilitate comprehensive preoperative survival assessment. The results of this study highlight the crucial role of basophil and PBR in the assessment of prognosis in GBM patients, and future studies should focus on the complex interactions between basophils and tumor cells in the tumor microenvironment to illustrate the mechanisms of basophils influencing survival.

## Acknowledgments

We thank Doctor Yi Guo for his help in the procedure of statistical analysis. We thank Professor John for his support in the language of the article.

## Author contributions

**Conceptualization:** Chao Yang, Zhiqiang Li.

**Data curation:** Jian Xu, Jie Wang.

**Formal analysis:** Chao Yang.

**Methodology:** Chao Yang, Qiong Yao.

**Supervision:** Qiong Yao.

**Writing – original draft:** Chao Yang.

**Writing – review & editing:** Chao Yang, Jian Xu, Qiong Yao.

## Supplementary Material


