# Supplementary material for: Prognostic value of platelet-to-basophil ratio (PBR) in patients with primary glioblastoma
Source: Medicine (Baltimore). 2023 Jul 28;102(30):e34506. doi: 10.1097/MD.0000000000034506 (PMC10378900; doi:10.1097/MD.0000000000034506)

Supplementary Fig. S1 Cut-off value of different blood markers in patients with glioblastoma: (A) EBR; (B) LBR; (C) LER; (D) NBR; (E) NER; (F) PER

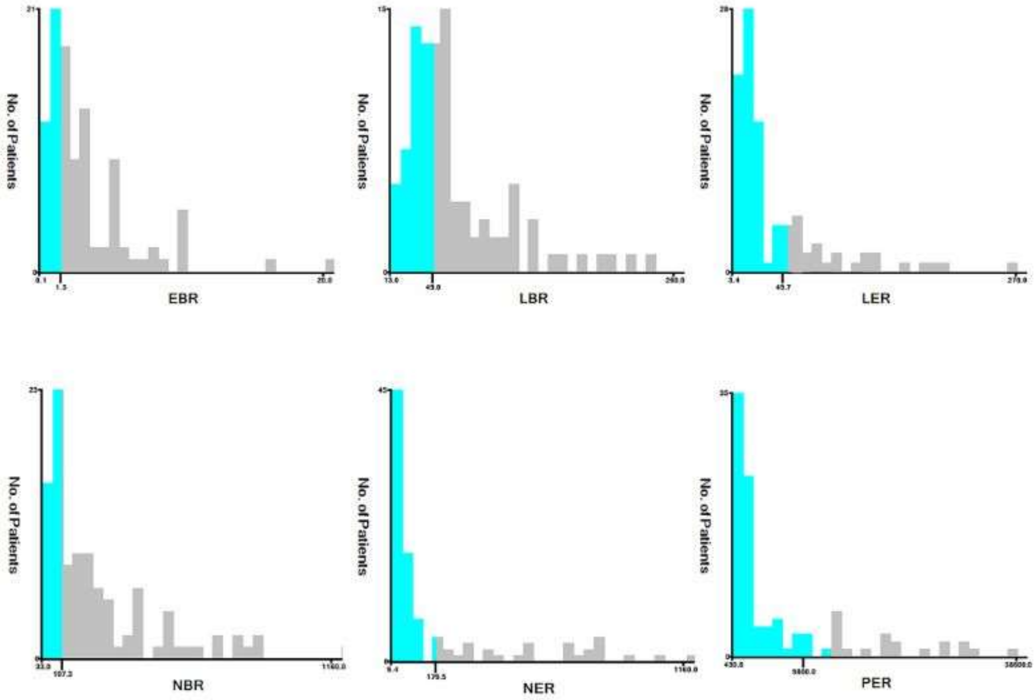

Supplement: Supplementary file 1 [file medi-102-e34506-s001.pdf]
